# A Compact and Low-Power SoC Design for Spiking Neural Network Based on Current Multiplier Charge Injector Synapse

**DOI:** 10.3390/s23146275

**Published:** 2023-07-10

**Authors:** Malik Summair Asghar, Saad Arslan, Ali A. Al-Hamid, HyungWon Kim

**Affiliations:** 1 Department of Electronics, College of Electrical and Computer Engineering, Chungbuk National University, Cheongju 28644, Republic of Korea; summair@chungbuk.ac.kr (M.S.A.); alihamid@chungbuk.ac.kr (A.A.A.-H.); 2 Department of Electrical and Computer Engineering, COMSATS University Islamabad, Abbottabad Campus, Univeristy Road, Tobe Camp., Abbottabad 22044, Pakistan; 3 TSY Design (Pvt.) Ltd., Islamabad 44000, Pakistan; saad.a@ieee.org

**Keywords:** spiking neural network, leaky integrate and fire, neuromorphic, artificial intelligence, artificial neural networks, Internet of Things, CMOS

## Abstract

This paper presents a compact analog system-on-chip (SoC) implementation of a spiking neural network (SNN) for low-power Internet of Things (IoT) applications. The low-power implementation of an SNN SoC requires the optimization of not only the SNN model but also the architecture and circuit designs. In this work, the SNN has been constituted from the analog neuron and synaptic circuits, which are designed to optimize both the chip area and power consumption. The proposed synapse circuit is based on a current multiplier charge injector (CMCI) circuit, which can significantly reduce power consumption and chip area compared with the previous work while allowing for design scalability for higher resolutions. The proposed neuron circuit employs an asynchronous structure, which makes it highly sensitive to input synaptic currents and enables it to achieve higher energy efficiency. To compare the performance of the proposed SoC in its area and power consumption, we implemented a digital SoC for the same SNN model in FPGA. The proposed SNN chip, when trained using the MNIST dataset, achieves a classification accuracy of 96.56%. The presented SNN chip has been implemented using a 65 nm CMOS process for fabrication. The entire chip occupies 0.96 mm^2^ and consumes an average power of 530 μW, which is 200 times lower than its digital counterpart.

## 1. Introduction

In an effort to make Internet of Things (IoT) hardware more intelligent, artificial intelligence (AI) is being employed in the next generation of IoT applications. The inclusion of AI in IoT promises new horizons while presenting some challenges. Existing AI neural networks use CPU/GPU hardware architectures, which are not feasible for IoT applications with limited power. IoT applications have scarce energy sources and thus require low-power solutions to ensure the longevity of the devices [1].

On the other hand, neuromorphic systems for prospective computing systems that mirror a biological neural network have received great research interest. These systems are highly energy-efficient and perform parallel signal processing [2,3]. In many IoT applications, deep neural networks (DNNs) are being recognized for achieving high accuracy of classification [4]. However, DNNs’ excessive calculations and the exigencies of memory over a conventional von Neumann computing system make them power-hungry and occupy more bandwidth. Thus, they are not applicable to IoT and mobile applications such as object recognition in drones [5].

In contrast with today’s digital microprocessors, the biological human brain is established on a non-von Neumann architecture [6], wherein neurons (processing elements) and synapses (memory) are collocated. The human brain consumes less energy (20 W) for computations at a higher speed [7]. Neuromorphic architectures mimicking the human brain have found feats in energy efficiency and can utilize a complementary metal oxide semiconductor (CMOS) to realize various computational models of neural entities [8]. Recently, spiking neural networks (SNNs), imitating biological neural networks, are becoming popular due to their parallel signal processing while consuming less energy [9]. SNNs realized in hardware can be very energy-efficient for the power constraints in IoT applications [10]. Moreover, SNNs benefit from high processing speed and imitate the temporal behaviors of the neuronal membrane [11]. Of these benefits, the SNN finds feats in various IoT applications with edge AI sensors. For example, IoT networks for image recognition tasks employ low-power sensors with embedded AI. Autonomous vehicles with edge AI perform high-speed image detection with edge AI sensors. Moreover, IoT networks with edge AI sensors are used for energy management and intelligent factories.

The implementation of a neural network on hardware by Carver Mead is credited with laying the foundation for a new school of thought in neuromorphic engineering. He demonstrated how analog systems, which are more robust to component degradation and failure and consume less power, may be used to emulate brain characteristics on hardware [2]. An integrated approach should be used, keeping in mind the limitations and difficulties that neuromorphic engineering is now facing. Better hardware may be produced by utilizing the benefits of technological innovation and scalability in the field of very large-scale integration (VLSI). Neural networks must be implemented as a system-on-chip (SoC) using a significant number of transistors that serve as processing and communication components. The scalability of CMOS allows for the compact construction of neural networks with high levels of integration. However, this still falls short of the biological neural network of the brain’s integration density. Due to this, several hybrid approaches have been researched, employing a combination of durable CMOS and novel devices such as memristors. Memristors crossbar architecture finds benefits in discrete devices and easier difficulties, whereas scalable and reliable CMOS-based architectures are still being researched for large-scale architecture development. Therefore, neuromorphic chips can be realized by benefiting from the robustness and scalability of CMOS [12].

In this paper, we present an analog CMOS-based hardware implementation of an SNN for IoT and mobile applications. The SNN SoC proposed in this paper is constituted of synapse and neuron circuits, optimized for the area and power consumption, which was presented in the prefatory prototype design [13,14]. The SNN architecture further improves the area, power efficiency, and accuracy at a small cost of increased complexity compared with the predecessor chip [14]. The SNN SoC is fabricated using a 65 nm CMOS process technology. The same SNN model has also been implemented in full digital implementation, so that area and power consumption can be compared with the analog SoC implementation. The remainder of this paper comprises the following sections. Section 2 presents the SNN and neuronal model along with the optimized architecture and design of the SNN. Section 3 introduces the analog circuit implementations and design techniques used to achieve compact and low-power circuits and demonstrates simulation results of the SNN in comparison with digital implementation. Then, CMOS-based analog and digital SNN implementations are analyzed, and measurement results of the hardware implementations are presented in Section 4. Moreover, in Section 5, the performance of the proposed SNN SoC implementation for various parameters is presented and compared with other state-of-the-art SNN SoC implementations. Finally, the conclusions are drawn in Section 6.

## 2. Neural Network Architecture

### 2.1. Spiking Neural Network Model

A third-generation SNN has been chosen in this work for the on-chip implantation of neural networks due to its brain-like achievements and efficiency in spatial–temporal coding [15]. An SNN is a network of neurons interconnected through synapses capable of performing inference and training tasks. The information delivery process in the SNN resembles a brain wherein interconnected neurons become activated upon receiving discrete input spikes that are evoked at different time intervals. A neuron in a layer receives input spikes from a neuron in a previous layer via synapses. The weight of the synapse modulates the incoming spikes and generates an equivalent current. The charge from all input synapses is accumulated in the form of potential on the neuron’s membrane. The neuron evokes an output spike when its membrane potential accumulates up to a predefined threshold value. Therefore, synapses can be considered as a memory with a communication interface, while neurons can be considered as a processing unit able to accumulate and compare.

### 2.2. Leaky Integrate and Fire Model

**Figure 1:**
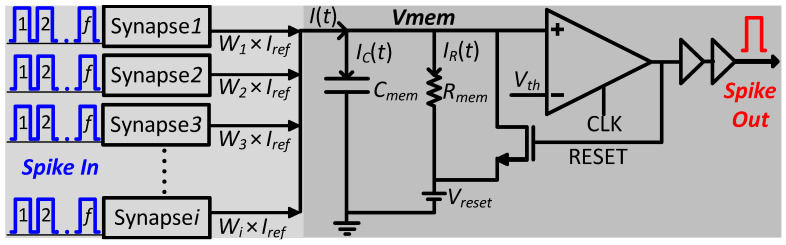
An LIF-based neuronal model of CMOS neuron cell.

The Leaky Integrate and Fire (LIF) neuronal model has been adopted for realizing the large-scale SNN in this work, as it encapsulates biological computational features of a neuron by integrating simpler circuits on silicon [16]. The robust CMOS-based LIF neuronal model finds advantages over other models [17] since it allows for a compact silicon implementation of a large SNN on a chip. A representation of the LIF neuronal model with elementary CMOS devices is shown in Figure 1. Here, the neuronal membrane potential *V_mem_* can be modeled as a parallel connection of the Resistor–Capacitor network. The response of the parallel structure of *C_mem_* (membrane capacitance) and *R_mem_* (membrane resistance) can be modeled by Kirchhoff’s current law [11] and is defined as in Equation (1).

(1) $$I\left(t\right)=I_{R}\left(t\right)+I_{C}\left(t\right).$$

Here, the synapse acting as a current source injects a current *I*(*t*) into the neuron, charging *C_mem_* with current *I_C_*(*t*) and discharging *C_mem_* through *R_mem_* (leakage path) with current *I_R_*(*t*). When *V_mem_* ≤ *V*th, input synaptic current from a multitude of input synaptic sources accumulates charge over *C_mem_* and increases *V_mem_*.

(2) $$I\left(t\right)=\frac{V_{mem}-V_{reset}}{R_{mem}}+C_{mem}\frac{\partial V_{mem}}{\partial t},$$

(3) $$C_{mem}\frac{\partial V_{mem}}{\partial t}=I\left(t\right)-\frac{V_{mem}-V_{reset}}{R_{mem}}.$$

When *V_mem_* ≥ *V*th, *V_mem_* triggers the neuron to evoke an output spike signal, and *V_mem_* becomes immediately reset to the resting potential *V_reset_*. Equation (3) defines *V*th as the threshold voltage value at which the comparator decides to evoke an output spike. *V_reset_* is the resting potential of the neuronal membrane after an output spike is evoked. The total current *I*(*t*) is the sum of all the excitatory and inhibitory currents injected by all the input synapses and is expressed as

(4) $$I\left(t\right)=\underset{i}{\sum }\underset{f}{\sum }W_{i}\times I_{ref},$$

Here, in Equation (4), *I_ref_* is the reference synaptic current to be generated upon receiving a pre-synaptic input spike *f* (the number of spikes). *I_ref_* is then multiplied with the weight *W_i_* of the *i*th synapse to generate the total injected current *I*(*t*) [14].

### 2.3. Optimization of SNN Architecture

The proposed SNN SoC architecture adopts a spike signal representation called Binary Streamed Rate Coding (BSRC), which has been introduced by [18]. It has been shown that BSRC allows for an optimized SNN model for compact hardware and employs direct training for high accuracy based on an off-chip training technique. This direct training technique uses the exact model of SNN hardware implementation for determining floating point weights by propagating spike signals in the form of binary streams through synapses and neurons in each layer of the SNN model. Afterward, the floating-point weights are quantized into integer weights having a minimum bit-width of 5-bit values, which are good enough to achieve the target accuracy of 96% or higher for the MNIST dataset.

**Figure 2:**
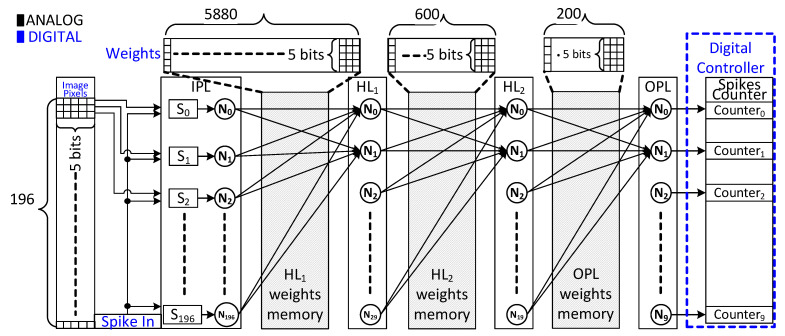
The architecture of the proposed SNN chip consists of four fully connected layers.

The overall architecture of the proposed SNN chip implementation is shown in Figure 2. The architecture, after optimization using the BSRC SNN model, is composed of four fully connected layers, namely (196-30-20-10), integrating synapse circuits, neuron circuits, and flip flops for storing image pixel and weight values. The first input layer (IPL) consists of 196 neurons that receive 196 individual grayscale pixels of the input image of size 14 × 14. The two hidden layers (HL1, HL2) consist of 30 and 20 neurons, respectively, while the output layer (OPL) of 10 neurons is used to classify each handwritten digit image of the MNIST dataset into ten numbers from 0 to 9. A total of 6680 synapses fully interconnect all the 256 neurons in each layer with one another. The 14 × 14 pixels of each image from the MNIST dataset are provided to the IPL synapses, which convert each pixel value into a stream of spike signals. As a result, the IPL generates 196 spike signal trains. Each spike train consists of spike pulses ranging from 1 spike up to 15 spike pulses. The proposed architecture distributes the weight memories to all the synapses. Small registers are collocated with associated synapse circuits in each layer. The register in the IPL stores a 5-bit pixel value, while the register in the remaining layers keeps the weight values. The proposed architecture, with distributed memory, benefits from minimal routing for high-speed processing and lower power consumption. The OPL consists of 10 neurons, which produce output spike trains. The proposed architecture has a digital controller (DC) that counts the number of spike pulses produced by each neuron of the OPL and classifies the image based on which output neuron gives the maximum spiking activity.

## 3. Implementation of SNN SoC

### 3.1. Analog SNN SoC Implementation

The four-layer SNN model in Figure 2 has been constructed from 6680 synapse circuits interconnecting different neurons. To keep size and power consumption low, a prototype synapse and neuron circuits were proposed earlier [13]. These circuits serve as a building block for our SNN hardware implementation in this work.

**Figure 3:**
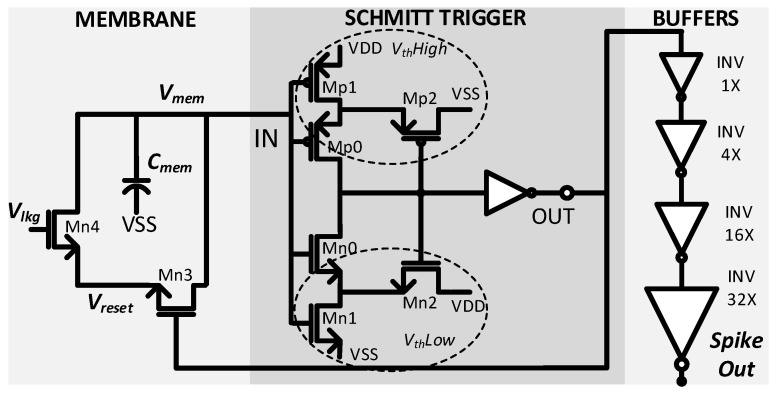
Proposed neuron circuit based on LIF.

The neuron circuit is established on the LIF model of the neuron, as shown in Figure 3. A Metal–Insulator–Metal Capacitor (MIMCAP) *C_mem_* of 10 fF is used to realize the neuronal membrane, which accumulates all the input synaptic currents to produce *V_mem_*. MIMCAP realizes *C_mem_* to ensure optimum linearity and minimal power consumption. The comparator, a crucial part of the LIF model, determines when to fire output spikes using a condition such as *V_mem_* ≥ *V*th.

The proposed asynchronous Schmitt Trigger circuit [13] serves as a comparator and benefits from optimum *V*th, high sensitivity, less area, and power consumption. The comparison of *V_mem_* and *V*th determines whether or not the Schmitt Trigger fires an output spike. When the Schmitt trigger fires an output spike, the feedback path resets the membrane potential to the initial potential (*V_reset_*). The leakage resistance and reset feedback path are implemented by NMOS constant current source and switches, respectively. Four output buffer stages are implemented, with an incremental 4× larger buffer in the next stage, to drive the synapses of the next layer. The neuron circuit in [13] offers a more optimal and compact structure than in [14].

**Figure 4:**
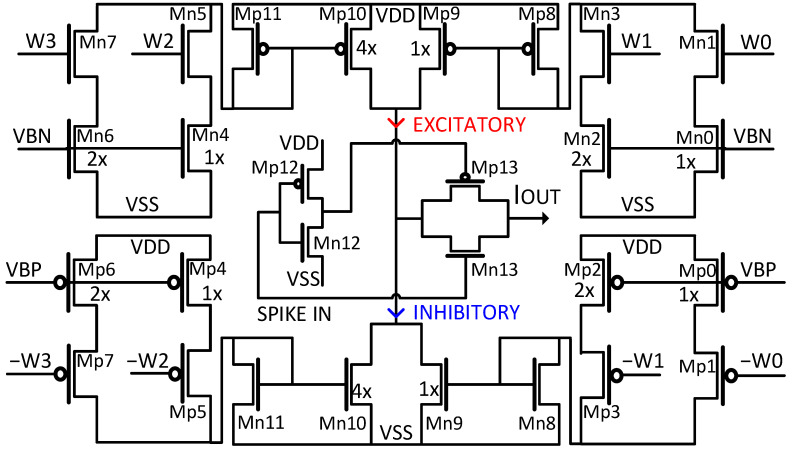
The schematic of the CMCI-based synapse circuit.

The synapse circuit designed for the proposed SNN, as pictured in Figure 4, is a 5-bit binary weighted current mirror structure named a “Current Multiplier Charge injector” (CMCI). The circuit embodies two symmetric and complementary portions. An excitatory portion, which is made up of four binary-weighted NMOS branches, and an inhibitory portion, which is made up of four binary-weighted PMOS branches. Each synapse calculates either a positive current amount modeling an excitatory action or negative current amount modeling an inhibitory action. Upon receiving an input spike event, the weight of each synapse determines the amount of current to be injected or ejected. The sign bit of the 5-bit weight parameter determines whether the synapse undertakes an excitatory or inhibitory action. Then, 4-bit LSBs of weight value determine the amount of binary-weighted current to be injected (excitatory) or ejected (inhibitory). The binary-weighted current is enabled by each weight bit connected to each branch of the synaptic circuit. The current mirroring transistors multiply the current of the right half by 1× and of the left half by 4×, thus forming binary-weighted currents by using four branches which are turned on or off by each of the weight bits, w_3_, w_2_, w_1_, and w_0_. The final current of the synapse circuit is accumulated on or ejected from the membrane potential capacitor. The splitting of the synapse circuit into symmetric right and left halves and the current multiplication reduces the size of the proposed CMCI synapse circuit by 60% compared to the conventional binary-weighted circuits. Moreover, the flip-flops are utilized in conjunction with each synapse circuit to store pre-trained weight values on synapses. The CMCI structure synapse consumes less area and power than the previous synapse circuit proposed in [14] while providing higher design scalability for higher resolutions.

**Figure 5:**
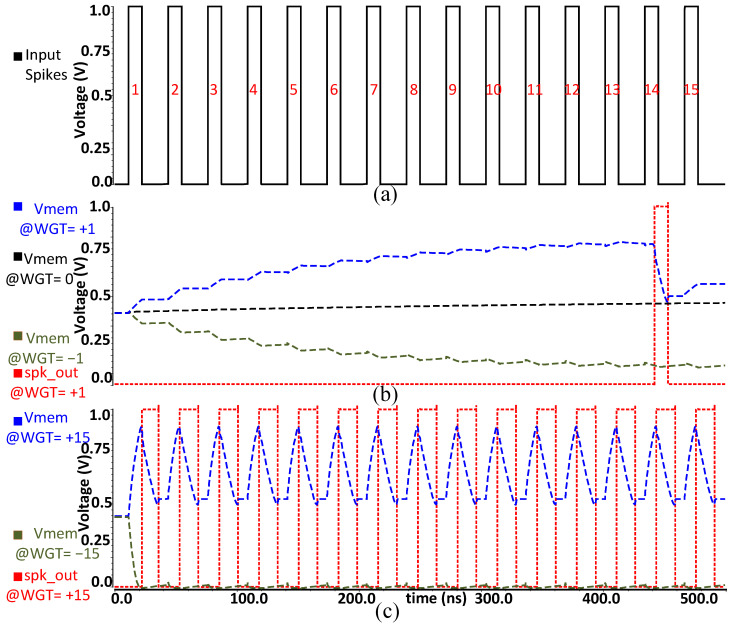
Simulation results for single neuron cell where (**a**) is the enable signal of 15 input spike signals, (**b**) *V_mem_* along with output spike for weight values +1, 0 and −1, (**c**) *V_mem_* along with output spikes for weight values +15 and −15.

One neuron cell comprising one synapse circuit and a neuron circuit was simulated, and the results are shown in Figure 5. It illustrates input spikes, accumulation of *V_mem_*, and evoking of output spikes for different values of 5-bit weights. For this simulation, the synapse circuit first receives a 5-bit weight parameter, which is pre-determined by the training process of the SNN system of our concern. As shown in Figure 5a, a sequence of 15 input spike pulses acting as an enable signal is supplied to the CMCI synapse. As can be seen in Figure 5b,c, for a synapse with an excitatory weight (MSB = 1), each input spike makes the generated current charge up *V_mem_.* Once *V_mem_* exceeds the pre-determined threshold *V*th, the neuron circuit fires an output spike. For example, if the weight value is +15, a single input spike generates the highest current amount, which rapidly charges *V_mem_*. On the other hand, for a synapse with an inhibitory weight (MSB = 0), each input spike makes the current discharge *V_mem_*. Therefore, the inhibitory weight refrains *V_mem_* from evoking an output spike. After the neuron evokes an output spike, it resets *V_mem_* to *V_reset_*. In case of either no input spike or a weight value of 0, *V_mem_* remains at the same value or decreases over time due to the leaky integration function of the LIF neuron.

### 3.2. Digital SNN SoC Implementation

In addition to the analog implementation of the SNN shown in Figure 2, we also implemented a full-digital design based upon the BSRC SNN model using Verilog HDL for the purpose of comparing the two implementations in terms of area and power consumption. Like the analog SNN implementation discussed above, the input layer of the digital SNN comprises 196 synapses to convert pixel values into spike signal trains. The digital SNN is constituted of multiple synapses connected to neuron logic that stores its membrane potential in its register memory. When an input spike arrives at a particular synapse, then its weight value is accumulated in the membrane register. The neuron evokes an output spike when the value in the membrane register reaches a threshold value and resets the value of the membrane register. A discrete spike-event strobe signal, which is produced by dividing the system clock by a known factor, triggers the operation of neurons and synapses at its rising edge.

## 4. Measurement Results

### 4.1. Analyzing Analog SNN

**Figure 6:**
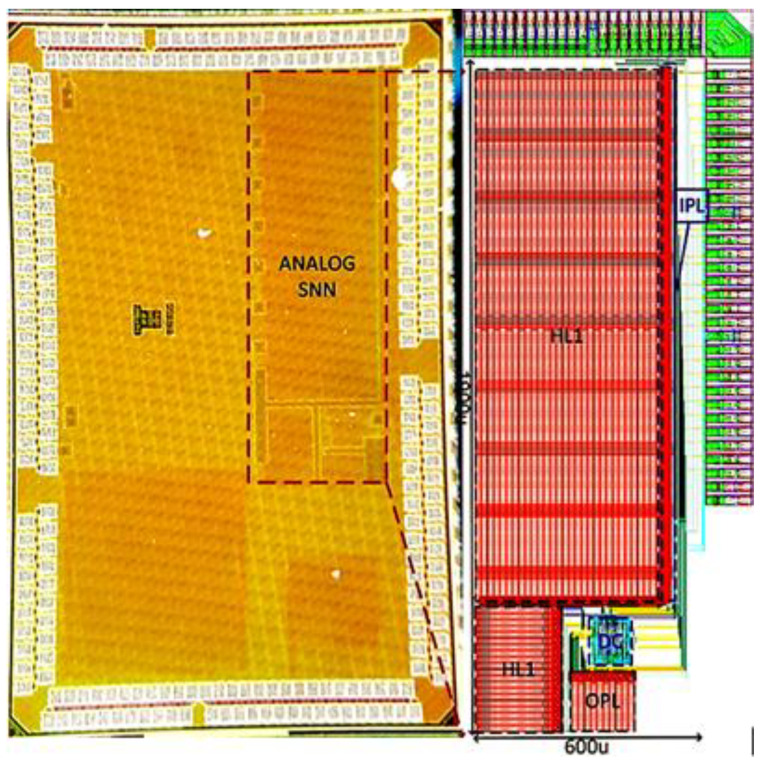
The micrograph of SNN chip with complete layout.

The proposed SNN architecture shown in Figure 2 was implemented and fabricated using a 65 nm CMOS process design kit. The entire chip layout of the analog SNN integrated with pads is highlighted and demarcated in the micrograph of the fabricated chip in Figure 6. All the four fully connected layers of the SNN are tagged as IPL (input layer), HL1 (hidden layer1), HL2 (hidden layer2), OPL (output layer), and a DC (digital controller). To minimize the chip size, all the layers are aligned abreast to curtail the routing, which leads to an analog SNN chip with an active core area of 0.96 mm^2^.

**Figure 7:**
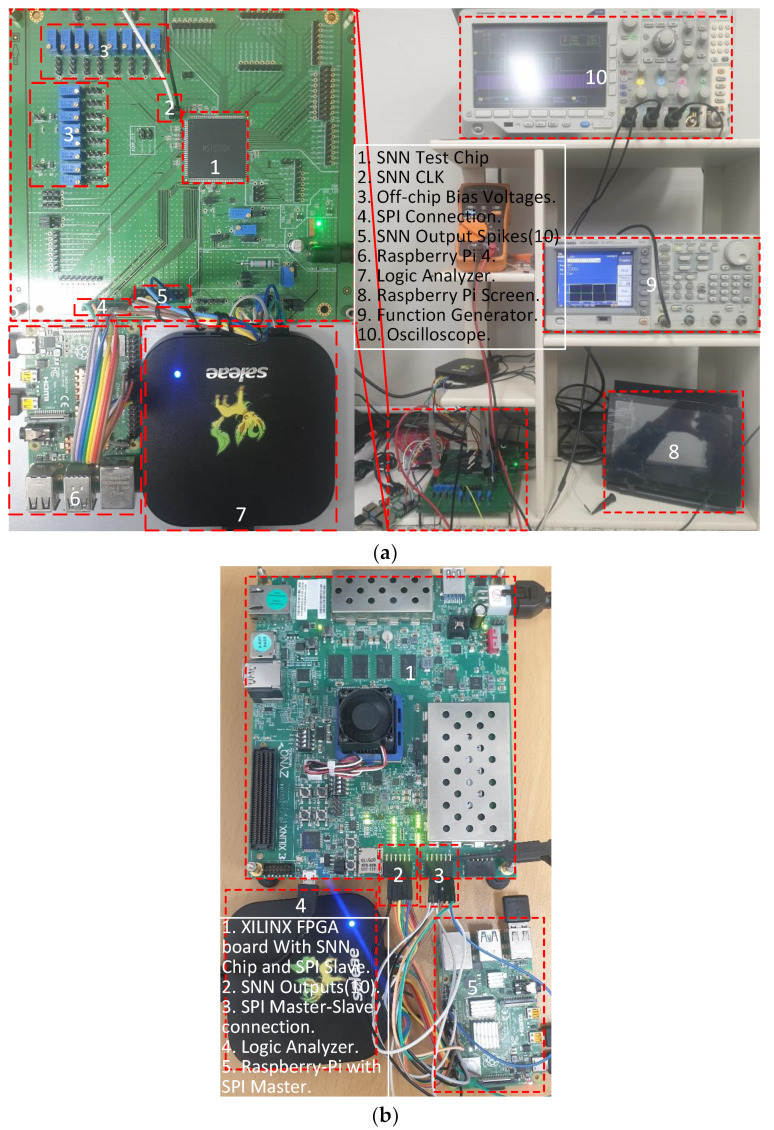
Measurement setup of (**a**) analog and (**b**) digital SNNs.

The measurement setup, along with the printed circuit board (PCB) for the fabricated analog SNN, is shown in Figure 7a. The input image, weight data, and configuration parameters are provided to the SNN chip by a host CPU board (in our measurement setup Raspberry Pi 4) via a serial parallel interface (SPI). Once configured, the on-chip digital controller (DC) takes weight values from the host CPU and stores them in the weight memories, which are connected to synapses. It then takes input pixel data and converts them into input spike signal pulses based on the BSRC coding described in [18]. Then, the spike signals propagate from the input layer IPL through each layer, eventually reaching the output of the output layer OPL. The DC counts the spiking activity of the ten outputs of OPL, converts it into a digital value, and forwards it to the host CPU board for further estimation of classification.

**Figure 8:**
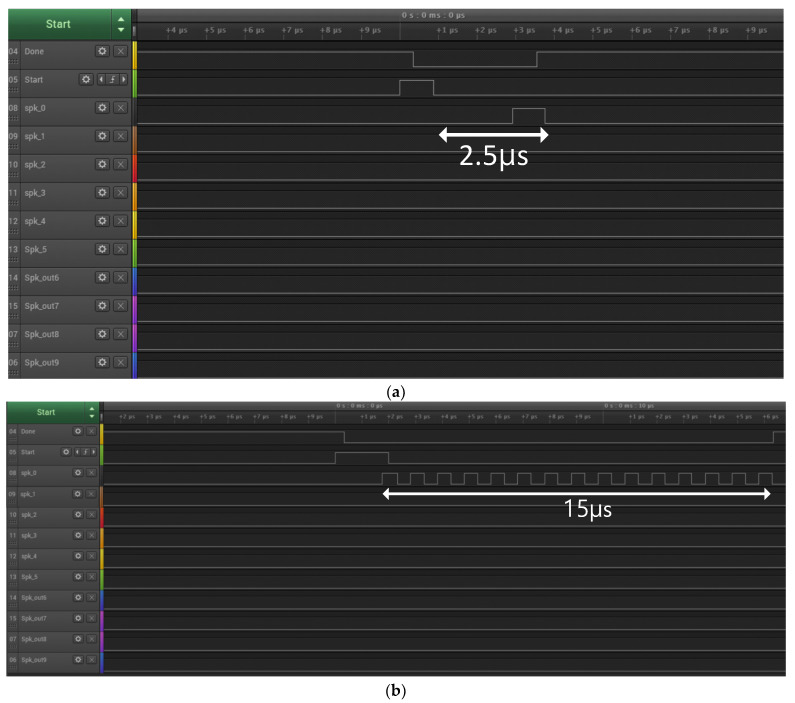
Spike signals of SNN chip measured by a logic analyzer: (**a**) One input spike signal propagation; (**b**) 15 input spike signals propagation.

The outputs of the SNN chip are also measured using a logic analyzer, as shown in Figure 8a,b. In Figure 8a, one input spike is propagated to the first output node through the first neuron of all four layers when the maximum weight value of 15 is written to the first neuron’s synapse of every layer and a weight value of zero to the rest of the synapses. Similarly, as shown in Figure 8b, keeping the same weight configuration as the former measurement, 15 input spikes are propagated to the first output node. These measured results demonstrate the successful spike propagation from the input layer to the output nodes of the fabricated analog SNN chip. The average power consumption of the analog SNN chip was 530 μW when measured using the MNIST dataset at a clock frequency of 10 MHz. We also calculate energy per spike, which is the total energy consumed for propagating all input spike events divided by the total number of input spike events. In the test of the proposed SNN chip shown in Figure 8a, which is operated at 10 MHz, a single input spike takes 2.5 μs for propagation, thus consuming 1.325 nJ of energy per spike.

### 4.2. Comparison with Digital SNN Chip

**Figure 9:**
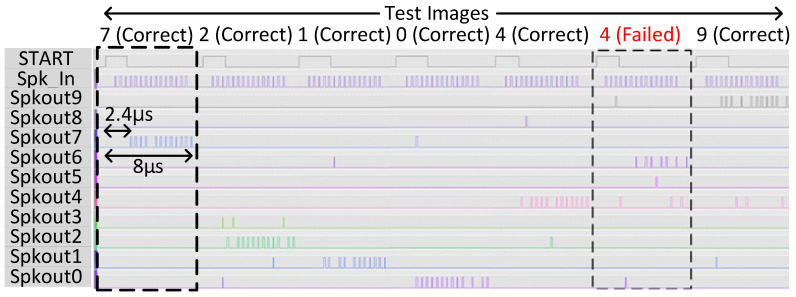
Measured classification result for digital SNN.

The full-digital SNN chip described in Section 3 has been implemented in FPGA for comparison purposes. The FPGA board is measured by employing the test setup shown in Figure 7b, wherein a host CPU board (Raspberry Pi 4) provides input image and weight values via an SPI. To test the digital SNN FPGA, the on-chip controller generates stimulus input spike signals in a sequence of digital pulses. The output spiking activities at the ten output nodes are forwarded to the host CPU for further classification, like the case of an analog SNN chip. These outputs of the digital SNN are taken out of the FPGA and observed via a logic analyzer, as shown in Figure 9. Here, the spiking activities of the ten output nodes are visualized for correct and failed classification against each input test image. The digital SNN occupies an area of 0.75 mm^2^ and a power consumption of 117 mW, which are estimated by Synopsys Design Compiler using the same 65 nm CMOS process as the analog SNN. From the measurement results of Figure 9, wherein the digital SNN operates at 10 MHz, a single input spike takes 2.4 us for propagation, thus consuming 4.660 nJ of energy per spike. Moreover, the digital SNN takes 8 us per image to complete, thus consuming 936 nJ of energy per image. From this, the energy per image for the analog SNN can be calculated as 261 nJ.

Table 1 highlights the comparison. While the analog SNN chip is slightly larger (28%) than the digital SNN chip, it consumes significantly less energy per spike and per image (72%).

## 5. Performance Analysis

To evaluate the performance of the implemented SNN chips, we chose the SNN model for the digit image classification task based on the MNIST dataset of handwritten digits. MNIST consists of 50,000 training and 10,000 test images. Using the BSRC encoding [18], we optimized the SNN model and trained it in a Python framework. Afterward, the SNN chip utilized these trained weights for inference using the MNIST dataset. The SNN hardware, in analogy to its software counterpart, attained the targeted average accuracy of 96.56% for classification.

Table 2 compares the performance of the proposed SNN chip with previous works [5,11,19] and its predecessor chip [14]. A figure of merit, namely “complexity”, is defined as the total number of weights, which allows for a fair comparison of the physical dynamics of different neuromorphic chips implemented for various applications. We then calculated the area and power efficiencies (η) by dividing the complexity (number of weights), respectively, by consumed area and power. The proposed SNN implementation outperformed the previous works [5,11,19] and its predecessor chip [14] in terms of area, power, and accuracy while consuming slightly more energy. The previous work [5] based on a radial basis function network (RBFN) with multilayer perception (MLP) incorporates a compact analog core to provide good area efficiency. It, however, suffers from poor power efficiency due to its recursive operations. The neuromorphic chip called HICANN-DLS [11] incorporates an array of neuron cells based on compact LIF with wide tunable parameters, which benefits from its low energy per spike of 790 pJ. Its total power consumption, however, is excessively high, leading to very low power efficiency (598 times lower than the proposed work). In addition, it shows significantly lower area efficiency (24 times lower than the proposed work). Compared with the predecessor work [14], the proposed work offers an improvement of 7.5 and 4.0 times in terms of area and power efficiency, respectively.

## 6. Conclusions

This paper proposes a spiking neural network SoC hardware implementation optimized for area and power consumption. The four-layer SNN is constituted of compact synapse and neuron circuits, occupies a die area of 0.96 mm^2^, and consumes 530 μW of power. The SNN chip successfully achieves a targeted calcification accuracy of 65.6% while consuming 1325 pJ of energy. The SNN can be easily extended for higher resolution and a number of classes. Therefore, making it a suitable candidate for mobile applications. It will also expand the IoT horizon to cover an even more comprehensive range of applications (which are currently deemed impractical), due to the improvement in the ratio of performance to power consumption.

## Figures and Tables

**Table 1:** Comparison of analog and digital SNNs.

S NN CHIP	Area	Energy/Spike	Energy/Image
Analog SNN	0.96 mm^2^	1.325 nJ	261 nJ
Digital SNN	0.75 mm^2^	4.660 nJ	936 nJ

**Table 2:** Proposed SNN chip comparison with other neuromorphic chips.

Parameters	[11]	[5]	[19]	[14]	This Work
CMOS tech [nm]	65	130	800	180	65
Architecture	Analog	Analog NNC	Mixed-Mode	Analog	Analog
Classifier Type	SNN	MLP/RBFN	SNN	SNN	SNN
Neuron Model	LIF	-	LIF	LIF	LIF
Neuron cell Area (μm^2^)	2352	68,400	-	2022.7	228
Neuron cell power (μW)	14.4	723	-	25	3
Chip Area (mm^2^)	3.6	0.140	1.6	3.6	0.96
Power (mW)	48.62	2.20	40 μ	1.06	530 μ
Energy per Spike (pj)	790	-	900	900	1325
Accuracy of MNIST [%]	-	92	-	94.60	96.56
Complexity (total # of weights)	1024	750	256	3311	6680
Area η (Complexity/Area)	284.5	5360	160	920	6958
Power η (Complexity/Power)	21.06	341	-	3123	12,603

## Data Availability

Not applicable.
